# Value Chains of Public and Private Health-care Services in a Small EU Island State: A SWOT Analysis

**DOI:** 10.3389/fpubh.2016.00201

**Published:** 2016-09-14

**Authors:** Sandra C. Buttigieg, Marcus Schuetz, Frank Bezzina

**Affiliations:** ^1^Department of Health Services Management, Faculty of Health Sciences, University of Malta, Msida, Malta; ^2^Aston Business School, Aston University, Birmingham, UK; ^3^School of Social Policy, College of Social Sciences, University of Birmingham, Birmingham, UK; ^4^Department of Management, Faculty of Economics, Management & Accountancy, University of Malta, Msida, Malta; ^5^Faculty of Business and Economics, Hong Kong University of Science and Technology, Kowloon, Hong Kong; ^6^Department of Organization and Management, Faculty of Economics and Business, University of Zagreb, Zagreb, Croatia

**Keywords:** value chains, public health sector, private health sector, Malta, SWOT analysis, small island state

## Abstract

The global financial and macroeconomic crisis of 2008/2009 and the ensuing recessions obliged policy makers to maximize use of resources and cut down on waste. Specifically, in health care, governments started to explore ways of establishing collaborations between the public and private health-care sectors. This is essential so as to ensure the best use of available resources, while securing quality of delivery of care as well as health systems sustainability and resilience. This qualitative study explores complementary and mutual attributes in the value creation process to patients by the public and private health-care systems in Malta, a small European Union island state. A workshop was conducted with 28 professionals from both sectors to generate two separate value chains, and this was followed by an analysis of strengths, weaknesses, opportunities, and threats (SWOT). The latter revealed several strengths and opportunities, which can better equip health-policy makers in the quest to maximize provision of health-care services. Moreover, the analysis also highlighted areas of weaknesses in both sectors as well as current threats of the external environment that, unless addressed, may threaten the state’s health-care system sustainability and resilience to macroeconomic shocks. The study goes on to provide feasible recommendations aimed at maximizing provision of health-care services in Malta.

## Introduction

Health is undergoing a paradigm shift from disease-focus toward systems-focus on a global level in response to both the financial crisis and in response to problems encountered in disease-specific projects in honoring targets and internationally agreed benchmarks (1). Governments are, therefore, increasingly scrutinizing returns on health-care expenditure and pushing health actors to reorganize inefficient and badly functioning public systems. There is an increasing need for a synergistic set of policies, which requires joint action of health and non-health sectors, of public and private actors and of citizens for a common interest. The 2008/2009 global financial and economic crisis, as well as the ensuing recessions, pushed governments to emphasize governance for health in the pursuit of a healthy workforce and health as vital to well-being through both whole-of-government and whole-of-society approaches (2, 3), so as to improve societies beyond economic development (4).

The challenges are greater for small states as these do not have a large portfolio of institutions to provide health-care services. Indeed, in a narrative literature review on policy challenges and reforms in small European Union (EU) member states, Azzopardi-Muscat et al. (5) conclude that “lack of capacity and small market size give rise to common challenges in governance and delivery of health services in small states” (p. 6). Therefore, so as to maximize the provision of health-care delivery as well as to ensure health system resilience, small states must endorse the active involvement of all institutions that are relevant to health-care delivery, irrespective of whether they are public or private entities. Dafflon and Vaillancourt (6) argue that competition between private and public hospitals/clinics does exist and is challenging for coordination of services. In view of emerging evidence that effective channels improve public hospital performance, some suggest that hospital competition should be nurtured (7). On the other hand, others argue that the strategies that promote competition may not lead to improved quality due to a dominating price competition, with purchasers and consumers preferring lower premiums at the expense of improved quality (8). Moreover, competition between providers may be asymmetric (9), in that, if the public sector provides excellent health-care delivery then the private health-care sector loses competitive advantage; if the private health-care sector is successful, then pressure on the public sector is reduced.

This empirical study aims to identify and analyze complementary and mutual attributes in the value creation process to patients by the public and private health-care systems in Malta, a small EU island State, through workshop settings involving a wide array of professionals from both sectors. Through an analysis of strengths, weaknesses, opportunities, and threats (SWOT), we explored the strengths and weaknesses of the internal environment and the opportunities and threats of the external environment of both sectors in an attempt to provide recommendations aimed at maximizing provision of health-care services in Malta. Collaboration between public and private hospitals/clinics was identified as a priority of the Maltese Government (10). Public–private collaboration has become widespread globally as an approach for improving all the dimensions of good quality in health-care delivery, as defined by the Institute of Medicine, namely safe, effective, timely, efficient, equitable, and people-centered care (11, 12), while aiming for health system financial sustainability and resilience to macroeconomic challenges.

## The Maltese Health-Care System

Malta is a EU member state and is an archipelago in the Mediterranean Sea consisting of three main islands, Malta, Gozo, and Comino. It is the most densely populated country with the lowest total population of any EU member state (13). Life expectancy compares well with the EU average and has improved over the past 20 years. In 2015, life expectancy at birth: for the total population was 80.3 years (compared with 80.2 for the EU); 77.9 years for men (compared with 77.4 years for the EU as a whole) and 82.7 years for women (compared with 83.2 for the EU) (14).

The Maltese health-care system is composed of the public sector, which is free at the point of use for all Maltese citizens and migrants residing in Malta, who are covered by Maltese social security legislation. Excluded from this entitlement are elective dental care, optical services, and some formulary medicines, which are means-tested as per the non-contributory scheme of the Social Security Act (Chapter 318 of the Laws of Malta) (15, 16). Individuals that fall within the low-income bracket are entitled to free medicines from a restricted National Health Service (NHS) formulary of essential medicines and to certain medical devices. In addition, chronic illnesses included in a specific schedule incorporated in the Social Security Act are covered by entitlement to free medicines related to the illness. This benefit is independent of financial means (15, 17).

The Maltese NHS, which is inspired by the British NHS, provides a comprehensive package of health services. While both the Maltese and British health systems boast of universal coverage, questions often arise on their long-term sustainability. Nevertheless, in view of Malta’s major focus on universal coverage, self-reported unmet need due to financial constraints was reported to be 0.8% in 2010 and, therefore, much lower than 2.3% European average (17). Running in parallel is the private sector, which accounts for a third of total health expenditure and provides the majority of primary care (17). The total health expenditure as a percentage of gross domestic product (GDP) in Malta was 9.7% in 2014 (18), while its GDP was ranked 17th out of the 34 European countries in the EQUI-HEALTH project and, therefore, at the median. This is below the EU average of 10% (18). Of this, a third is private spending (2.9% of GDP, compared with 2.3% in the EU); public spending was only 5.6% of GDP, below the EU average of 7.3%. 66% of the financing comes from general tax revenues and other government sources, while 32% is provided by out-of-pocket payments (19). The health sector has to compete with other public sectors for funding from the Consolidated Fund of taxation revenue. Most of the out-of-pocket payments are to primary care practitioners (mainly GPs) in private practice, who account for two-thirds of primary care facilities in Malta. These services offer more personalized care as well as shorter waiting times and waiting lists. In recent years, the increase in private spending has outpaced public health expenditure growth (17). Table 1 provides an overview of the relevant health expenditure indicators of Malta as compared with EU-28, EU-15, and World Health Organization (WHO) European Region. Malta possesses relatively high out-of-pocket payments in view of the dominant private primary health care. However, only about one-fifth of the population purchase commercial health insurance policies (15).

**Table 1:** **An overview of Malta’s health expenditure indicators as compared with EU-28, EU-15, and WHO European Region (2012)**.

	Malta	EU-28	EU-15	WHO European region	Comments
Total health expenditure as % of GDP	9.1	6.4	10.4	8.4	The total health expenditure of Malta as% GDP has been steadily increasing since 2000
	9.6^a^				
General government health expenditure as a % of total government expenditure	13.2	11.8	16.1	13.2	The Maltese government expenditure rising steadily since 2000, with peak in 2007 for construction of new general hospital
General government health expenditure as a % of total health expenditure	67	72.5	77	69	Malta registers lower percentage than other averages in view of the dominant private primary health care
Public sector health expenditure as a % of GDP	5.5	7.3	8	5.5	Malta registers lower percentage than EU in view of the dominant private primary health care
Private household’s out-of-pocket payments on health as a % of total health expenditure	32.6	16.3	14.4	24.2	Malta possesses a relatively high out-of-pocket payments in view of the dominant private primary health care
Private household’s out-of-pocket payments on health as a % of private health expenditure	93.8	84.1	62.5	74.8	Malta registers higher percentage than other averages in view of the dominant fee-for-service private primary health care

*Source: Ref. (16)*.

*^a^2014*.

*EU-28: the current 28 EU Member States: Austria, Belgium, Bulgaria, Croatia, Republic of Cyprus, Czech Republic, Denmark, Estonia, Finland, France, Germany, Greece, Hungary, Ireland, Italy, Latvia, Lithuania, Luxembourg, Malta, Netherlands, Poland, Portugal, Romania, Slovakia, Slovenia, Spain, Sweden, and the United Kingdom*.

*EU-15: Austria, Belgium, Denmark, Finland, France, Germany, Greece, Ireland, Italy, Luxembourg, Netherlands, Portugal, Spain, Sweden, and the United Kingdom*.

*WHO European Region: Albania, Andorra, Armenia, Austria, Azerbaijan, Belarus, Belgium, Bosnia and Herzegovina, Bulgaria, Croatia, Cyprus, Czech Republic, Denmark, Estonia, Finland, France, Georgia, Germany, Greece, Hungary, Iceland, Ireland, Israel, Italy, Kazakhstan, Kyrgyzstan, Latvia, Lithuania, Luxembourg, Malta, Monaco, Montenegro, Netherlands, Norway, Poland, Portugal, Republic of Moldova, Romania, Russian Federation, San Marino, Serbia, Slovakia, Slovenia, Spain, Sweden, Switzerland, Tajikistan, The former Yugoslav Republic of Macedonia, Turkey, Turkmenistan, Ukraine, United Kingdom, and Uzbekistan*.

Table 2 provides an overview of relevant health resource indicators for Malta as compared with EU-28, EU-15, and WHO European Region. What is most striking is the shortage of nurses and also shortage of curative beds as compared with European and Organisation for Economic Co-operation and Development (OECD) averages. Indeed, bed occupancy rate for the main acute general Mater Dei Hospital in Malta has been mostly over 85% since it opened in 2007 (17). One of the great challenges facing the Maltese health-care system include overcrowding of Accident and Emergency Department, which has become a bottleneck in view of problems with having patients admitted to hospital (20). Furthermore, the hospital faces the challenges of discharging older patients who require long-term care and rehabilitation (21).

**Table 2:** **An overview of Malta’s relevant health resources indicators as compared with EU-28, EU-15, WHO European Region, and OECD (2012)**.

	Malta		EU-28	EU-15	WHO European region	OECD		Comments
Number of all practicing doctors per 100,000 population	329		345	352	340	n/a		The number of practicing doctors in Malta is slightly less than European averages
Number of all practicing nurses per 100,000 population	668		803	836	722	n/a		Malta has consistently suffered from shortage of nurses since 1990s, and the number of practicing nurses is much below European averages
Number of all practicing midwives per 100,000 population	40		33	32	44	n/a		Malta compares well with European averages as regards number of practicing midwives
Nurses and midwives:physicians	02:01		2.4:1	2.4:1	2.3:1	n/a		Malta registers lower nurses and midwives:physicians ratio mainly due to shortage of nurses
Availability of diagnostics and therapeutic infrastructure per 100,000 population	MRI: 0.72	CT: 2.86	n/a	n/a	n/a	MRI: 1.39	CT: 2.37	Malta has better CT than MRI coverage as compared with OECD
Number of curative hospital beds per 100,000 population	257		385	350	510	334		Malta has a shortage of curative beds as compared with European and OECD averages

*Source: Ref. (16)*.

*OECD: international organization helping governments tackle the economic, social, and governance challenges of a globalized economy. Australia, Austria, Belgium, Canada, Chile, Czech Republic, Denmark, Estonia, Finland, France, Germany, Greece, Hungary, Iceland, Ireland, Israel, Italy, Japan, Korea, Latvia, Luxembourg, Mexico, Netherlands, New Zealand, Norway, Poland, Portugal, Slovak Republic, Slovenia, Spain, Sweden, Switzerland, Turkey, United Kingdom, and the United States*.

Other challenges facing the Maltese health-care system include progressively aging population, with direct influence on the public finances’ sustainability; the growing burden of non-communicable and chronic diseases; as well as financial and infrastructural limitations. In addition, Malta has, for recent years, invested poorly in organized public health and prevention programmes. Indeed, the last reported comparative percentages of health budget allocated to organized public health and prevention programmes show Malta at 1.3% compared with EU – 24 at 2.9% (22). Furthermore, the determinants of health as specified in the Health System Performance Assessment, in particular, the proportions of the overweight and obese, are deteriorating (16).

Over the past decade, the Maltese Government has collaborated with the private sector, in particular to tackle waiting lists in several medical imaging and surgical interventions, namely for PET, MRI and CT scans, cataracts, total knee replacements, and total hip replacements (10).

## Porter’s Value Chain Model

In his original approach to analyzing value chains, Porter (23) uses a rather fixed framework of operational steps in which value is created in a company to achieve competitive advantage. He describes the value chain as the internal processes/activities a firm performs “to design, market, produce, deliver, and support its product” (p.33). Activities are split into a sequential stream of activities, and this facilitates the identification of primary activities (inbound logistics, operations, outbound logistics, marketing/sales, and service) and support activities (procurement, technology development, Human Resource Management, and firm infrastructure). Although Porter’s value chain model has had its fair share of criticisms (24, 25), there have been multiple modifications and adaptations of the original approach [e.g., see Benjamin and Wigand (26) and Sharma et al. (27)], as well as methodological evolvement of practical tools, so as to apply the analysis to non-production entities and also parts of the operations, like logistics [as in the case of the study by Hines et al. (28)]. For health care, as a service industry, the foundation of applying value chain analysis has been laid by the Wharton School study (29). The Wharton School Study of the Health Care Value Chain is based on the US private mix health-care system with its unique composition and stakeholders that characterize this system. Our study provides separate value chains for the public and private sectors of the Maltese system, where the state is the main owner of the health-care system supported by parallel private services. Hence, the value chains illustrated in this study specifically reflect the two sectors of the Maltese health-care system with the described compositions and stakeholders.

## Methodology

We invited a group of 30 health-care professionals from different disciplines with experiences ranging from 5–35 years (medical administrators, medical doctors across specialties, dentists, nurses, physiotherapists, occupational therapists, and laboratory scientists) from both the public and private sectors to participate in a workshop. Twenty-eight health-care professionals responded to the call: 16 and 12 contributed to the public and private sector workshops, respectively. A brief was emailed to all participants beforehand stating that we were holding a workshop: (1) to analyze the value chains and business models of public and private health-care service providers in Malta, (2) to identify strengths and weaknesses in each step, and (3) to see whether there are complimentary factors. The participants were also provided with background reading on value chains in health care.

The participants were again briefed at the beginning of the workshop on the theory, in particular based on Porter’s value chain model. In the setting of this paper, the value chain has been derived by members of two workshop groups, and according to the operation purpose, without having a strict framework given with minimal intervention from the researchers. The advantage of this approach is, that new aspects, which are, value creating can be added and potential efficiency improvement or cooperation of different industry participants can be identified. In diversion from Porter’s (23) general value chain structure, as a first step, the workshop participants were requested to define the sequence of value creation in their sector. On the basis of this value chain, the participants were asked to define perceived strengths (S), weaknesses (W), opportunities (O), and threats (S). It is worth noting that participants from both groups at times failed to distinguish between the internal and external environment during the SWOT analysis; some modifications were, therefore, necessary during transcribing to ensure that the final versions of the value chains were in line with the definitions of SWOT. The setting and briefing was conducted in a classical way so as to enable analysis of competitive advantages and shortcomings in the two sectors. Participants were also informed of our intention to investigate factors of maximizing the provision of health-care services in Malta. One aspect of this, was to stimulate group discussions on whether there is an optimum for the mix of public and private service providers and how the market and interaction should be ideally structured to provide overall highest service levels.

After the joint introduction and briefing, the facilitated workshop sessions were conducted in separate rooms at the same time, whereby the participants were separated on the basis of their experience. The groups first defined the purpose and target of their operation, separately as an orientation point for the discussion. During the sessions, there was no interchange between groups. The two groups’ discussions triggered participants to contribute toward drawing the value chain for the public and private sector on large charts. This enabled active participation by all participants. Once the groups’ workshops ended, the two groups joined into one and listened to each group leader interactive presentation. The presentations of the two group leaders and the discussions that ensued were audio-recorded and transcribed verbatim. All the data (value chains drawn in workshops; final presentations of workshop leaders) were analyzed as part of the SWOT analysis in the value chains for the public and private hospitals.

## Results

The following are the salient findings emerging from the workshop. The operation purposes of the two groups were defined as:

Public healthcare provides vital healthcare services to the citizens of Malta, maximizing the quality of care by an efficient use of resources. (Group on Public Sector)

Private healthcare in Malta maximizes shareholder value by selling healthcare and related services (Group on Private Sector)

### Public Sector Workshop Session

**Figure 1:**
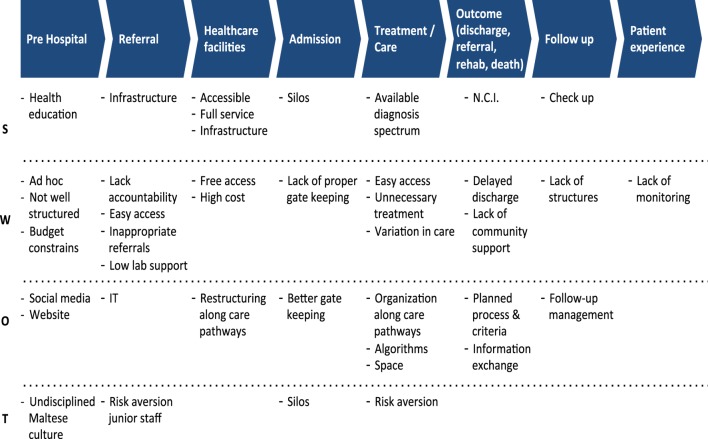
**Value Chains of Public Health Care (Mater Dei Hospital and Health Centres)**.

Figure 1 illustrates the emerging value chain for the public health-care sector in Malta. This value chain starts off with pre-hospital care, leading to referral to secondary/tertiary care, health-care facilities, hospital admission, treatment/care of inpatients and outpatients, outcomes of hospital care (discharge, referral, rehabilitation, or death), hospital follow-up, and ending with patient experience. The group recognized the importance of patient experience in the value chain:

We thought about patient experience, because we want to go from quality outcome to quality experience, which is even more important to some extent - less patient complaints.

#### Strengths

One of the major strengths of the public sector is its ease of access, universal coverage, is free at the point-of-use to not only Maltese citizens and migrants with the social security contribution but also to asylum seekers, and is accessible every day and hour of the week.

First, not the hospital but the free hospital because everybody on the national insurance contribution on the island can access it 24/7 … we even offer free service to asylum seekers

Health care facilities: we talked about hospital and free hospital, we said our strengths are that it is accessible

Other strengths identified were the public hospital’s location in the center of the island and that patients can get all the medical services within the same location.

It is right in the middle of the country … there’s a good infrastructure – it’s a one-stop hospital – everything in the same site, which is a big advantage so patients do not have to move from one place to the other.

The public sector as part of the civil service structure is able to address human resources needs and recruit health-care professionals accordingly.

There is a significant drive to have HR. There is no hesitation with having more staff, just a question of the availability of the staff but there is never a big issue that we do not want to employ the staff we have.

The government health service infrastructure also provides a link between the various services along the continuum of care from primary to long-term care/rehabilitation.

An advantage of the public sector is that the hospital, health centres, other public hospitals, as well as long-term care and rehabilitation all fall under the Department of Health with possibility of referral or communication. There is an infrastructure for referral.

The public sector organizes its own primary health-care centers for gatekeeping and follow-up following discharge.

The public sector can organize gatekeeping using the health centres – which are also free! The health centres support the hospital when patients are discharged for example removal of sutures …

The public sector’s infrastructure enables it to offer detailed investigations and state-of-the art medical imaging techniques.

The free hospital provides very good pathology and medical imaging services – which are considered to be state-of-the-art.

The public sector’s infrastructure enables it to focus on health promotion/health education.

So with free hospital … there might be a case to discuss prevention as well, before patients get sick, so we talked about medical free hospital education, set-up of education, screening … a lot of education and a lot of it being done ad hoc.

With regard to outcomes, the public sector recognized the hospital’s growing emphasis on patient safety and decrease in nosocomial infections.

As regards strengths of the outcomes in the value chain, we discussed PasQiT- the patient safety and quality improvement team which is active on safety alert learn, patient identification, near misses etc … more infection control … we have decreasing infections

The workshop participants highlighted the importance of having discharge liaison nurses to facilitate discharge of patients and to organize follow-up.

Discharge is more organised – patient discharged by his own nurse

#### Weaknesses

On the other hand, the participants identified several weaknesses ranging from contextual and structural problems to variation in the standards of care and unnecessary treatment/over-investigation/waste.

Easy accessibility (may be too easy), unnecessarily investigation abuse, variation in care between different areas, wards, sectors.

The public sector group recognized the challenges involved in providing all services for free at the point-of-use, concluding that the public sector is loss-making.

Cost-effectiveness versus profit. We talked about what is more important here, essentially. We are a loss-making industry all the time.

The group recognized that a weakness of the public sector is the lack of distinction between regulator, provider, and purchaser, resulting in lack of objective judgment across the supply chain:

We thought that there is a problem essentially because the provider and the purchaser are across the whole board, are the one and the same entity, to some extent, and I think that this was a significant weakness.

As regards referral to public hospital, the group identified easy access could be the result of lack of accountability in primary care.

We thought lack of accountability, access might be too easy for referral and people self-refer, they can go to GP and they are automatically referred, so, inappropriate referrals is a problem

The group emphasized that both providers and patients may abuse the free at the point-of-use public sector because of the lack of awareness of costs.

It is completely free, so patients do not appreciate what they are getting to some extent and we do not appreciate what we are giving, to some extent.

There is lack of cost consciousness and lack of quality culture. We need to make people more aware of what things cost because we do tests, patients get tests, and nobody knows what they are costing really.

As regards discharges, the public sector group recognized that the support from primary care/community is not optimal, in particular due to the absence of patient registration with GPs. This weakens the hospital’s follow-up structure; indeed hospital professional members of staff in Malta have to assume more post-discharge responsibilities in patient care than in other countries.

The late discharges, lack of community support … it is weak compared to other countries … lack of follow-up.

Absent patient registration in primary care, which renders hospital communication with identified GP more difficult

#### Opportunities

The group also identified several opportunities emanating from the external environment, namely use of social media and information technology; the impact of patients’ lobby groups on policy makers to secure better resources for specific diagnostic groups; the use of gatekeeping by private GPs and public primary care; and use of standard operating procedures, protocols, guidelines as part of integrated care pathways system-wide.

There were some opportunities for us that we could use social media, we could have a better website, informative website for free hospital care.

Referrals to secondary care or back to primary care, based on appropriate feedback, currently feedback is at times inappropriate. Discussed the possibility of having an IT system for referral … to be referred to the right places – right referral, right attendance.

The group recognized the importance of gatekeeping to avoid unnecessary load on the main hospital, with suggestions for specialized hospital staff to assist health centers with achieving this:

We have better gatekeeping, outpatients, health centres, maximize gatekeeping and improve gatekeeping.

We discussed the possibility of people from the hospital working in health centres in the community because that would give a better opportunity at gatekeeping and better education of other gatekeepers.

The group identified the public hospital as a teaching hospital with strong links to the Medical School and the Faculty of Health Sciences at the University of Malta. This arrangement should be exploited better to build multidisciplinary/interdisciplinary teams.

Our Medical School and our Institute of Health Care [now Faculty of Health Sciences], same University where we work together.

Most of the staff here know each other, work together for a long time, so doctors know each other (same medical school) … nurses and other staff, same nursing school … and we should be using that to an advantage to a greater amount, having more multi-disciplinary work. There have been some improvements, I think, with this.

The group also discussed that the different aspects of care should be linked through organization of care pathways and development of algorithms. This is possible in the public sector if the departments have integrated IT systems and proper channels of information exchange.

Opportunities to have care pathways, so that we would have a plan which is not standardized for each department, but a plan for everybody involved in care of what is going to happen … and use of algorithms.

#### Threats

The public sector group identified several threats in the external environment, namely Maltese culture that still shows lack of discipline as regards misuse of public service. The somewhat “abusive” Maltese culture is characterized by high number of self-referrals to emergency department for the lower priority ailments. For example, patients show up at A&E with problems that could have easily been managed in the primary care sector.

We talked about the culture of the population which is potentially a threat … people abuse the system because it is free

Similarly, the lack of discipline is reflected when some patients who are booked for outpatients and to a lesser extent surgical procedures simply do not turn up on the day, without informing the hospital. This results in waste of time space and resources in particular when the public sector suffers from long waiting lists.

Another threat is the no shows for outpatient appointments and to a lesser extent procedures … at times you have patients who resort to the private sector fed up with waiting … then do not inform the hospital

Other threats include risk aversion by GPs resulting in more referrals to hospital:

There is also risk aversion by GPs, especially since GPs are not always supported by investigative back-up … at times it is easier to pass on the problem to the hospital.

If the GP has asked for an MRI because he is risk avert, as a consultant I will do the MRI because if I don’t, it will just carry on.

Other threats mentioned by the group include aging population resulting in more older adults – often with more chronic diseases need to access hospital; and lack of long-term care/rehabilitation beds creating several bottlenecks – at A&E, and acute hospital care. The group also mentioned cultural challenges from having an increasing migrant population.

A threat to our hospital is the changing demographics, ageing population

For example going to the Obstetrics department. We have gone from a 4% foreign clients … we are now 17% foreign clients, within 10-15 years … that might be a threat which we really need to look at - problems with communication and cultural differences.

The group also mentioned of the health system as a threat, whereby long-term goals may be jeopardized in preference to attaining vote-catching short-term results.

Unfortunately, Malta being small tends to have politicization of many issues … including health!

We talked about the issues from the politicians, and these are the ones who complain most … they seem to have the most leverage on what happens and what change happens …

#### Silos

Finally and interestingly, participants mentioned silos several times, namely as a strength of the public service in terms of the pride shown by specific disciplines but also as a weakness in that silo mentality adopted by disciplines fragments care and causes duplication of work.

Silos … can be a strength actually because people who work in silos are actually proud of the service that their department is providing, but at the same time at the expense of what another silo is providing. So for the patient we have to look at what is best.

There are silos, which is a weakness for some issues … everyone fights for his own territory, for his own department, when in actual fact we should be fighting for the same patients.

“Silos” is also identified as a threat of the external environment in that different sectors in the public service may not always work together to solve health issues. For example, collaboration between health and education, health and legal system, and health and social policy are some of the multi-sectoral approaches that would benefit the health sector in the long-term.

### Private Sector Workshop Session

**Figure 2:**
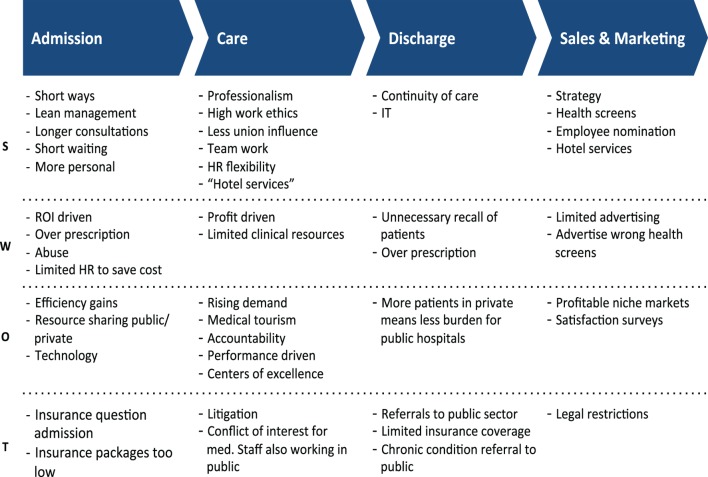
**Value chains of private health care**.

Figure 2 illustrates the emerging value chain for the private health-care sector in Malta. The value chain includes four main parts, namely admission, care, discharge, and sales and marketing.

#### Strengths

A major strength as identified by the group is the short wait time to reach the specialist of your choice, who provides more personalized attention and care, as well as devotes more time per consultation as illustrated by workshop private sector session group leader.

So while the public have months on end waiting, here within a few days, if not the following day, you are going to be seen, if not the same day … there is less bureaucracy … you get a better consultation … there is continuity of care

Another strength is the greater ease with which team of different professionals can communicate with each other in the private as compared with public sector a stated below:

The team as a hospital is going to communicate with each other, so the physiotherapist will discuss with the dermatologist to know where the problem is because this is a smaller hospital and they can all interact much better together.

We all know how these multidisciplinary teams work, not only at a small hospital there is more harmony with these teams working together and all this is exchanged into patient’s satisfaction and benefit to the patients.

The group mentioned lean management as a strength in maximizing efficiency while ensuring low waste. However, the group insisted that the private sector is market-driven and focuses on services that ensure a good return on investment as stated below:

The investment which is required in a private field depends on the market. So if I am the manager, this is market driven. I am going to invest into an analyser or particular equipment if this is going to give me profit. If it’s not, then this will be referred to the public … there could be a hidden agenda

The private sector makes sure that the patients are satisfied with care as well as offers optimal “hotel services” within the limits of the cost insured, and this triggers the private sector to be efficient:

We know that there are gaps in the insurance because it is a limited amount … this hospital has limited resources so you have to be really efficient

To get a patient satisfied with what you are giving with these less resources we have to be more efficient … so that drive is healthy always to improve, so that we change the standards which are required by these insurance companies.

The group recognized that the professionals in the private sector are all covered by private indemnity insurance, seem to show high work ethic, and are able to work flexibly and in a team. There is also training on attaining standards on patient care.

Every health care worker in the private hospital is trained on how to care for the patient. Before they are certified as compliant of patient care, they are not allowed to go in the ward next to the patients, so there is much more individual attention.

In this regard, another strength is the private sector’s ease of recruitment and selection in contrast to the public sector:

One important thing which I love in the private sector is to hire and fire human resources … if you are not efficient, they will tell you: thank you very much, don’t turn up for work the following day.

In a bid to achieve client satisfaction and aiming for a “return-customer” policy, the private sector ensures continuity of care and follow-up.

There is going to be certainly, an assurance of continuity of care or follow-up. We use electronic reminders, that they have to follow up … they get better discharge notes, the prescription is more easy to read (medicines), etc.

As regards sales and marketing, the group identified this part of the value chain as very strong with the aim of attracting clients.

The Marketing Strategy usually is decided by the Management Board. It is usually very efficient, up-to-date … you get all Marketing … if you go on Facebook, if you go on television, you are going to find Marketing.

The private sector tends to emphasize having highly motivated staff and excellent hotel service that ensures privacy and comfort in their marketing messages.

The employees are more motivated … they are going to give a better service to their customer, that once we give a hotel-comfort service, everyone is happy and smiling.

The private sector group also mentioned that, over the years, there has been more interest in offering health screening packages, for example, breast screening albeit the free mass screening as a free service that was introduced by the Government.

There are health screens, which are promoted by the private entity and this is beneficial to the patient because it promotes a healthy population.

#### Weaknesses

Of concern is the mention of potential abuse (and, therefore, waste of resources) in terms of over-prescribing, prescribing more expensive drugs, and over-investigating or using the more expensive medical imaging techniques so as to increase profit, all of which may not be needed if diagnosis and treatment can be reached at lower cost. According to the group, this attitude by the private sector seems to be more salient when caring for privately insured patients.

Also, there could be abuse in the system. I could over prescribe … tests, medicines etc. because I am going to make more profit and also could be insurance driven

The group suggested that since the private sector is profit-driven, esthetics might come before rational decisions in terms of patient safety.

If you look at your room in the private hospital you have got the carpet, though many say they shouldn’t be there because of infection control … but it looks nice. Your own colour TV … so there is luxury.

The group also mentioned that because of the focus on profit making, the private sector might resort to unnecessary recall of patients and prolonged length of stay:

There is the temptation to call the patient again. You might not wish to call him at such an early stage but you do … and this of course gives more profit.

There is also the tendency for a prolonged length of stay. In the public hospital, we are having problems with beds and trying to discharge … while there we can add another bed for a hundred Euro.

As regards sales and marketing, the group reiterated that despite the legal restrictions on advertising of services, the private sector advertises services and screening based on the extent to which these could provide a good return on investment.

The weaknesses about sales and marketing … first of all, it is limited by law, advertising and branding. By medical council, you are not allowed to do everything, but at the same time the private hospital could advertise their own ad screens, which are often chosen on the basis of profit.

Another weakness emanating from the public sector having training programmes for all health-care professionals and specialization routes:

There is lot of risk aversion, so especially if it’s junior doctors, junior members of staff … if you are seen by a senior member of staff that is fine! But if a junior member of staff sees you then if there is a problem, it’s always ‘because you did not consult your senior … ’ – so that induces risk aversion by the juniors

#### Opportunities

The group recognized that the collaboration between the public and private sectors can result in win–win situations for both sectors, in particular if resources are shared. The use of the private sector happens either through patients directly accessing the sector or through outsourcing from the public sector often as part of waiting lists initiatives. Therefore, the rising waiting lists and waiting times of the public sector due to increased public demand are opportunities for the private sector as the public can negotiate outsourcing terms.

There is outsourcing from public to private … if people go private they are not going to use the public facility … so the Government is going to spend less … but at the same time if the public is outsourcing, the private is making more profits, so this is a win-win situation.

Another opportunity for the private sector is optimal usage of technology in particular the use of automation that would save on human resources.

One has to invest in technology, it is very important especially with automation. The more automation you have, the less human resources required.

Another opportunity mentioned by the group is medical tourism, albeit still limited. The group identified dentistry as being successful in this area:

Medical tourism, as yet little effect on economy. We have mentioned dentistry as making in-roads

The group also identified profitable niche markets often in consultation with insurance companies who might be the ones to trigger introduction of new services based on clients’ demands.

Insurance companies approve services [if] there is an increase demand for a service … so the private hospital very quickly innovates to introduce that service - the customer is happy and the private hospital is making more profits.

#### Threats

The group acknowledged that the greatest threat for the private sector is financial sustainability. Clients tend to expect more from the private sector once they are directly involved in the fee transaction for the service, in contrast to the public sector which is free at the point-of-use.

The operation for private hospitals … is driven by economic prosperity in the country. There is more litigation and suing because the patient has forked out money so he expects more, so more legal problems.

The threats are mainly coming over here from finance. If you can’t afford our service you have to go public.

In particular, there may be interference from insurance companies on the management of care so as to fit the budget.

There might be insurance questions in management of care plan which might change … not really the most suited for our patients because we try to mould this according to the financial package and the patient might have to fork out more money to get all the services

A threat to the private sector is that when patients develop serious illnesses or complications, there often needs to be referral to the state hospital, which is more equipped in particular on the provision of intensive care services.

Discharge referrals to public sector. We always say, [Maltese expression – translated literally ‘when we find the bone’]‘fejn hemm l-ghadma’ … we send them to the public hospital, because the private hospital is not that equipped as the public.

During the joint discussion, the following salient points emerged on maximizing health-care provision for Maltese citizens through the involvement of both public and private sectors. Beyond maximization of efficiency in the public health-care sector regarding the management of the flow along patient pathways, and other efficiency measures, the combination of all health-care providers (public and private) should improve health-care provision for Maltese citizens. The group agreed that the existence of the two sectors in parallel to each other is necessary for Malta’s health-care system.

It is a good thing that we have the private sector, as I see it, as a gatekeeper, because if we do not have the private sector, I can assure you that the [public] hospital will collapse

The workshop identified that collaboration between the public and private sectors can help alleviate problems with physical space in the former.

We talked about physical space, even though we have such a big footprint on the hospital, we are still stuck with not enough space for a lot of departments.

Interestingly, the workshop participants acknowledged the already existent closeness between the two sectors, which could be exploited.

In Malta the healthcare, the agenda of public and private are so intertwined that the public is confused … so I am working here this morning and I am going to work in a private clinic in the afternoon, so what is my agenda?

So, if I give a good service here, I will get better return in my private sector but not everybody has the same agenda.

## Discussion

The value chain of the public health-care sector as defined by the workshop participants is far longer and overarching than the value chain for the private health-care sector, since this was identified to start before hospital admission – termed as pre-hospital care (GPs, screening, education, social care, etc.). The major reasons for the longer value chain were that public health care provides universal coverage of all health issues of the Maltese population, directs patients through specific diagnostic-based pathways, and offers fully centralized service facilities (17). The findings of this study with regard to the differences between the public and private health sectors are in line with the literature (1, 9, 12), namely the client-centered resources, management skills, comfort and technology of the private sector, and the regulatory actions and protection of the public interest of the public sector.

While the public sector uses money to provide health care, the private sector uses health care to make money. Both sectors strive for efficiency to achieve their goals. The incentive systems of the public and private health-care sectors are quite diverse with different strategies to achieve their respective operation targets (1). The public sector deals with aspects of health-care delivery that the private sector may not be directly interested in. For example, the provision of health education to the public at large, namely, with the aim of battling common unhealthy lifestyle issues of the Maltese population, is considered to be a priority of the public sector (17) and health insurance companies, since these stakeholders are paying for health care. Furthermore, prevention of disease would result in lower volume of demand on the public sector. In contrast, the private sector is more likely to invest in health checks and screening packages that would lead to diagnosis of disease, higher client volume, and demand for profitable procedures.

The three dimensions that characterize public entitlement to care are universality (population benefiting from public entitlement), comprehensiveness (public benefit packages that include full spectrum of services and across the continuum of care), and completeness of care in all categories (30). The public sector aims for patient satisfaction through quality, free access, and availability. The participants also claimed that the public sector referral process at primary health care improves gate keeping. This is in line with the European Observatory HIT report (17). However, primary health care seems to suffer from low laboratory and medical imaging support for *in situ* diagnosis, resulting in GPs taking a defensive attitude and potentially resulting into hypothetically avoidable referrals to secondary care. The other elements of the value chain concentrate on quality of care and efficiency. The risk and practice of waste in the public sector that includes over-investigation is acknowledged. The medical service portfolio of public health care includes high volume with diverse cost items.

The private sector aims for quality and client satisfaction to secure return on investment and shareholder value. This is in line with the findings in the literature (9, 12). This sector focuses on maximizing profitability by providing high quality health care and hotel services. The workshop participants concluded that this sector is not a competitor of the public health-care system, because it can take pressure from the public sector by providing beside medical services also convenience items (e.g., “Hotel services” and shorter waiting lists/times). The risk and practice of medically unnecessary patient recalls and over prescription is, nevertheless, acknowledged. The medical service portfolio of private care includes low volume, high cost, and high-cost items.

Neither the public nor the private sectors on their own are capable of solving the complex and abundant problems that health systems worldwide are facing (12). Therefore, so as to achieve a win–win situation for both sectors, any form of collaboration should be balanced and should be beneficial to both. This would entail structuring the market in a way that would maximize the provision of health care. There are three options of how this can be achieved so as to ensure a healthy and collaborative competition between the two sectors.

In our opinion, the most salient option for Malta is to maximize provision in having a regulated semi-competitive health-care model, which gives profitable services to the private sector, while at the same time ensuring a diminished volume of patients’ demand on the public sector. In this option, the public and private sectors with diverse goals coexist side-by-side without having to enter into complicated boardroom deals. However, the Government must ensure its capability in negotiating the setting of cost prices. Examples include laboratory and medical imaging services and collaboration in inventory management. This option would ensure market mechanisms and efficient patient flows in private and public clinics. This option might be supported by policies whereby citizens are encouraged to invest in private health insurance by introducing tax rebates. Indeed, the Maltese Government was at one point considering this measure (31); however, it never came to fruition.

A second option is the public–private mix model, whereby “private financing of care can make universal entitlement to care more ‘comprehensive’ and ‘complete’” [(30), p.1]. This option, namely of privately acquired entitlement with the public entitlement at the point of service provision can, however, interfere with the social goals of the public sector. The Maltese health-care system is similar to the British system; both having as their basis the principles of equity, universality, and solidarity. Therefore, should this option be utilized, the comprehensiveness and completeness of care should ensure coverage of the population’s health.

The third option is public–private partnerships (PPP), which are becoming more common in Europe, albeit mixed experiences (12), whereby Governments avoid capital outlay in joint ventures, while allowing the private sector to operate efficiently and to have a return on investment (32). However, the major disadvantage of adopting PPPs is that governance would be stifled by conflicting sectorial agendas, namely maximization of profit versus social needs that may render achieving direction and consensus difficult.

## Conclusion

This study has provided for the first time a comparison of the value chains of the public versus private health-care sectors in the small island state of Malta. The SWOT analysis revealed several strengths and opportunities, which can better equip health policy makers in the quest to maximize provision of health-care services. Moreover, the analysis also highlighted areas of weaknesses in both sectors as well as current threats of the external environment that, unless addressed, may threaten the state’s health-care system sustainability and resilience to macroeconomic shocks (33).

Two major recommendations emerge from this research. The first is investing in further public health education to combat lifestyle-related illnesses. The Maltese prevention is often more cost-effective in improving health rather than health spending once diseases takes root. There should be full cooperation with schools, fitness, and sports entities, while using specialized marketing agencies to ensure effective campaign. Budget constraints and ineffective coordination are restricting such activities. The second recommendation is to promote medical tourism, an activity seen as highly beneficial for small island states and developing countries (34). Additional revenues might be pooled to increase laboratory capacities, which are serving both the public and private sectors. Medical tourism should also contribute by increasing the volume of specific diagnoses in a small country like Malta that is required for specialists’ accreditation. This would be of benefit to the public health-care sector in Malta, while also potentially decreasing the number of patients requiring treatment overseas. However, we recommend that a policy paper for Malta is developed similar to the one by Lunt et al. (35), who developed conceptual frameworks for UK medical tourism, namely “Medical tourism pathways” (p.4) and “Framework for understanding medical tourist flows” (p.16).

Some limitations of the study should be noted. First, the study does not include the perceptions of patients and clients and, hence, we recommend that further research considers this major stakeholder of health-care service. Second, the contribution of pre-hospital primary care was identified by the public sector workshop, but not by the private sector one. This might be attributed to the perception of a higher percentage of patients accessing private specialist care directly, while bypassing primary care. Nevertheless, this finding and the fact that the length of the two value chains was different warrant further investigation. Third, so as to ensure maximum participation of a critical case purposive sample (36), namely the most suitable health-care professionals related to both sectors, the workshop was limited to 2 h. The time limitation may have limited the participants from exploring other points in the SWOT analysis, albeit encouraging them to be more focused. Fourth, this qualitative study was conducted in a single geographic location (Malta). Although islands have been used as small-scale laboratories for more complex politics of larger countries (37), we cannot rule out socioeconomic, political, and cultural biases. The findings of this study might not lend themselves to generalization over other cultures and societies. In this regard, this study needs to be backed up by studies in other jurisdictions with complimentary public and private health care, and using different research methodologies. Such research will generate more information than is currently available and will better advise health-care policy.

## Ethics Statement

Ethical permission was sought and obtained from the Research Ethics Committee of the Faculty of Economics, Management and Accountancy, University of Malta upon reassurance that the study did not involve vulnerable groups, such as the elderly, minors, or migrants, nor did it discuss subjects, which are sensitive in nature or involve the collection of personal data, such as political beliefs or religious opinions. Preceding the initiation of data collection, authorization was obtained from the Chief Executive Officer of the hospital, where the workshop was conducted. A cover letter reassured confidentiality and participant anonymity, while emphasizing that the participants had the ability to withdraw from the study at any time without any explanation. The principal researcher’s contact number was also included.

## Author Contributions

The three authors SB, MS, and FB contributed equally in the study design, conception, and drafting of the manuscript.

## Conflict of Interest Statement

The authors declare that the research was conducted in the absence of any commercial or financial relationships that could be construed as a potential conflict of interest.
